# THE EXISTENTIAL CONTEXT OF GRAY DIVORCE

**DOI:** 10.1093/geroni/igac059.154

**Published:** 2022-12-20

**Authors:** Torbjörn Bildtgård, Peter Öberg

**Affiliations:** Stockholms University, Stockholm, Stockholms Lan, Sweden; University of Gävle, Gävle, Gavleborgs Lan, Sweden

## Abstract

In later years divorce rates for older people has increased in many parts of the Western world in what has been described as a “grey divorce revolution”. In Sweden divorce rates for people 60+ have more than doubled since the millennium. The purpose of this paper is to study the reasons older people attribute to their late life divorce. Qualitative interviews were carried out with Swedish men and women aged 62–82 who after the age of 60 had divorced from a cross-gender marital or non-marital cohabiting union (n=37). The interviews covered themes regarding the divorce process, including reasons for divorce, experiences of divorce and life as a grey divorcee. The results were analysed using principles from Grounded Theory. The results revealed four different types of divorce narratives: 1) Incompatible goals for the third age, 2) Personality change caused by age related disease, 3) A last chance for romance, and 4) Enough of inequality and abuse. A central insight generated by the study was the importance attributed to the particular existential conditions of later life in the divorce decisions. The results are discussed in relation to theories of the third age as a time of self-fulfillment, where the partner can either be part of or an obstacle to that project.

